# Variability in Aggressiveness of Rice Blast (*Magnaporthe oryzae*) Isolates Originating from Rice Leaves and Necks: A Case of Pathogen Specialization?

**DOI:** 10.1371/journal.pone.0066180

**Published:** 2013-06-11

**Authors:** Abhijeet Ghatak, Laetitia Willocquet, Serge Savary, Jatinder Kumar

**Affiliations:** 1 Plant Breeding, Genetics and Biotechnology Division, International Rice Research Institute (IRRI), Los Baños, Philippines; 2 Department of Plant Pathology, G.B. Pant University of Agriculture & Technology (GBPUAT), Pantnagar, Uttarakhand, India; 3 Unité Mixte de Recherche Agrosystèmes et Agricultures, Gestion de ressources, Innovations & Ruralités (UMR1248 AGIR), Institut National de la Recherche Agronomique (INRA) and Institut National Polytechnique de Toulouse (INPT), Castanet-Tolosan, France; University of the West of England, United Kingdom

## Abstract

Rice blast, caused by *Magnaporthe oryzae*, causes yield losses associated with injuries on leaves and necks, the latter being in general far more important than the former. Many questions remain on the relationships between leaf and neck blast, including questions related to the population biology of the pathogen. Our objective was to test the hypothesis of adaptation of *M. oryzae* isolates to the type of organ they infect. To that aim, the components of aggressiveness of isolates originating from leaves and necks were measured. Infection efficiency, latent period, sporulation intensity, and lesion size were measured on both leaves and necks. Univariate and multivariate analyses indicated that isolates originating from leaves were less aggressive than isolates originating from necks, when aggressiveness components were measured on leaves as well as on necks, indicating that there is no specialization within the pathogen population with respect to the type of organ infected. This result suggests that the more aggressive isolates involved in epidemics on leaves during the vegetative stage of the crop cycle have a higher probability to infect necks, and that a population shift may occur during disease transmission from leaves to necks. Implications for disease management are discussed.

## Introduction

A few important pathosystems involve infections and injuries on different organ types. This is the case for example in diseases affecting leaves and fruits (e.g., powdery mildew and downy mildew on grapes, powdery mildew on strawberry, and apple scab), or diseases affecting leaves and storage organs (e.g., rice blast, wheat *Septoria nodorum* blotch, southern corn leaf blight). This may have important implications, because the types of organ injured correspond to different damage mechanisms, leading to different crop losses, both from the quantitative and qualitative standpoints. For example, injury associated to lesions on leaves may mainly cause a reduction in the green leaf area index (LAI), whereas infections on fruits or storage organs may directly reduce quantitative or qualitative harvests (i.e., crop losses). The analysis of the relationships between epidemics occurring on two organ types, or dual epidemics [1], is important to better understand the mechanisms underlying the quantitative and dynamic interplay between infections occurring on both organ types, and therefore to provide a sound basis for disease management.

In the case of rice blast, caused by *Magnaporthe oryzae* [2] (anamorph, *Pyricularia oryzae*), infections can occur on leaves, collars, necks, and panicles [3], [4]. Epidemics on leaves often occur mainly during the vegetative stage of the crop cycle, whereas the most important injury during the reproductive stage is associated to neck blast [5]. Epidemics on leaves generally correspond to a rapid increase of disease until maximum tillering, followed by a sharp decline, and a very low disease level from flowering until the end of the crop cycle. Neck blast lesions typically start to appear at flowering, therefore after epidemics on leaves have occurred. Yield losses associated to neck blast are much higher than yield losses associated to leaf blast in the tropical rice lowlands of Asia [5], [6].

Relationships between leaf and neck blast have been partly documented, and many questions remain. Although quantitative resistance against leaf blast is positively correlated with quantitative resistance to neck blast, some cultivars may be relatively resistant to the disease on one organ type and relatively susceptible on the other [5], [7]. The differential expression of resistance depending on organ may be explained by the existence of different types of resistance which expression could depend on organ, and/or by the adaptation to the type of organ infected within the pathogen population. In that latter case, a differential aggressiveness of isolates according to the organ they infect could be hypothesized.

While blast injuries occur on different tissues (leaves, collars, panicles, and necks), blast harmful effects (crop loss) increase in the same order as these four organs. Despite this, by far, the bulk of the research on blast has been focusing on leaf blast. This is because it is so much easier to infect leaves, often on seedlings, and analyze molecular processes associated with this type of injury. This begs the question of whether the large amount of (molecular) information accumulated that pertains to this type of injury can truly be extrapolated to other blast injuries. In particular, one may ask whether the genetic make-up of the pathogens causing leaf, collar, neck, and panicle blast are truly the same. A first step in this direction is to compare leaf- and neck- blast associated isolates, from a phenotypic standpoint. The objective of the work reported here was therefore to test the hypothesis of adaptation of *M. oryzae* isolates to the type of organ infected. To that aim, we assessed the effect of isolate origin (leaves on necks) on aggressiveness of *M. oryzae* on leaves and necks by measuring aggressiveness components on both types of organs.

## Materials and Methods

### Ethics Statement

The field sampling did not involve endangered or protected species. No specific permission was required for the sampling of rice leaves in fields, because IRRI is allowed to collect field samples as long as this does not cause any damage to the field.

### General Approach

Cross inoculation of *M. oryzae* isolates originating from lesions on leaves and necks was carried out to assess the effect of origin of isolates on their aggressiveness on leaves and necks. Two series of separate experiments were conducted, using the same pool of isolates, originating from leaves or necks. In the first series of experiments, the isolates were inoculated on leaves, while in the second one, isolates were inoculated on necks. For both series of experiments, aggressiveness was assessed by measuring parameters representing the different stages of the disease monocycle, or monocyclic variables [8].

### Plant Material

The tropical lowland rice variety IR50, susceptible to both leaf and neck blast [5] was used in all experiments. For leaf production, seeds were sown in a tray (26×34 cm) filled with soil and watered, and seedlings were grown for two weeks at 25–35°C inside a glass house. Nitrogen was supplied as ammonium sulphate (40 kg N ha^−1^) at 6 and 12 days after sowing.

For neck production, 2-week old seedlings, grown as described above, were transplanted (2 seedlings per hill) in a screen house at a 20×20 cm spacing. Nitrogen was supplied at 40 kg ha^−1^ at transplanting. Plants at panicle exsertion stage were transferred in pots (17.5-cm diameter) and placed inside a glass house until the end of the experiment. Two data loggers (Hobo, Onset Computer Corporation) were positioned inside the experimental area of the glass house to monitor temperature and relative humidity. The average temperature and relative humidity between inoculation and final assessment were 30.5°C and 76.6%, 30.6°C and 75.2%, and 29.5°C and 80.9% for the first, second and third experiments, respectively.

### Collection and Monospore Isolation of Isolates from Rice Fields

Samples of infected rice leaves and necks were collected in March 2010 in rice fields nearby the town of Cavinti (14°14′38′′ N, 121°30′35′′ E), Laguna Province, Central Luzon, Philippines. Samples were collected from four rice farmers' fields at flowering stage, each field being half to four kilometres away from one another. In each field, 21 infected leaves and 21 infected necks were sampled at random, the sampling locations being at least 5 m apart from each other. Samples were placed on a double layered moist filter paper in Petri plates, labelled, and stored at 4°C until processed for monospore isolation.

Infected samples were incubated in plates on moist filter paper in dark for 24 hr at 25°C in order to obtain sporulating lesions. Conidia from a sporulating lesion were transferred onto a 2% water agar plate, and incubated at 25°C for four to five hours. Individual germinated conidia were then transferred each into a slant with prune-agar medium and incubated at 26°C for five days [9]. Conidia produced from each slant represented a monospore isolate. Three isolates originating from leaves and three isolates originating from necks from each field, producing dark and fast growing colonies, were retained for this study. Therefore, a total of 12 isolates originating from leaves (LO-isolates) and 12 isolates originating from necks (NO-isolates) were used.

### Inoculation and Incubation Condition

#### Inoculation on leaves

The first fully expanded leaves on the main culm from two-week old seedlings were used. Four leaf segments (5 cm long) were placed (adaxial surface upside) on a double layered moist filter paper (Whatman no. 1) in a Petri plate. For each isolate, an inoculum suspension was prepared and adjusted to 5×10^4^ conidia ml^−1^ in a 0.025% Tween20 solution. Inoculum was sprayed as a fine mist using an atomizer consisting in a nozzle attached to a portable air compressor.

Four plates with leaf segments and one plate with 2% water agar medium were inoculated with 10 ml of conidial suspension for each isolate. The plate with water agar was used to estimate the density of deposited spores as well as their percentage of germination. These five plates were placed over a rotating tray that was rotated at 12 rpm to ensure a uniform distribution of inoculum. The nozzle of the atomizer was washed with 70% ethyl alcohol and distilled water before inoculation of another isolate. Inoculated plates were covered with their lids and incubated at 25°C with a 12 hr photoperiod provided by three cool white fluorescent lamps (36 W), installed at 50 cm height.

#### Inoculation on necks

Four necks per pot having reached the flowering stage were selected for inoculation. Leaf sheath was spread open in order to access the inoculation site, that is, the base of the neck. For each isolate, a conidial suspension was adjusted at 5×10^4^ conidia ml^−1^ of 0.5% gelatine solution. Each neck was inoculated with a 0.5 ml suspension by injecting the suspension into the base of the neck, using a 5-ml syringe. Inoculation was performed in the late afternoon in order to provide a cooler environment during inoculation and initial stages of infection. Inoculated plants (necks) were incubated in a glass house.

### Measurement of Aggressiveness Components on Leaves

#### Determination of leaf segment area and spore density

The width of all leaf segments was measured before inoculation. The leaf segment area (LSA, cm^2^) corresponding to leaf segments inoculated by a given isolate was calculated by multiplying the average leaf segment width by the leaf segment length.

For each isolate, the inoculated plate with water agar was incubated under dark for 4–5 hr at 25°C. After incubation, a drop of lactophenol cotton blue was dropped on two cover slips (1.8×1.8 mm each), which were placed onto the water agar. Conidia were counted on both cover slips using a stereomicroscope (×50 magnification), and the density of spores deposited, DSD (nb spores cm^−2^), was computed as the average over both cover slips of the number of spores deposited divided by the cover slip area.

#### Infection efficiency

Infection efficiency is defined as the ratio between the number of lesions and the number of spores deposited on leaf segments [8]. The number of lesions was counted at 5 day after inoculation (DAI) on leaf segments and infection efficiency was estimated as:
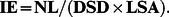



Where NL is the number of lesions counted per leaf segment, DSD is the density of spores deposited, and LSA is the leaf segment area.

#### Latent period

Latent period is the time elapsed between spore deposition and the beginning of sporulation [8]. The leaf segments in the plates were observed twice a day under a stereomicroscope (×50 magnification) starting 3 DAI. Here, latent period is operationally defined as the delay required to observe 50% sporulating lesions (LP50). This was estimated by linear interpolation of the number of sporulating lesions counted between successive assessments.

#### Lesion size

Lesion size was estimated from measurements of lesion length and lesion maximum width, using a ruler, at 5 DAI. The size of a lesion was estimated as 0.5×lesion length×lesion width [10].

#### Sporulation intensity

For all lesions produced, a portion of green leaf segment containing the lesion was dipped in a centrifuge tube containing 1 ml of 0.025% Tween20 solution. The tubes were shaken with a Vortex shaker (model: VM-2000, Digisystem Laboratory Instruments Inc., Taiwan) at full speed for 10 s to dislodge the conidia. The number of liberated conidia from a lesion was counted with a haemocytometer under a microscope (×40 magnification). Most assessments were made at 5 DAI. However, a few spore suspensions were stored at 4°C and spores were counted the following day. The mean number of spores over two counts was computed for each lesion.

### Measurement of Aggressiveness Components on Neck

#### Disease incidence

Although neck blast incidence, i.e., the number of infected necks divided by the number of inoculated necks, is not a direct measurement of infection efficiency *per se* [11], it is hypothesized that neck blast incidence reflects at least in part the efficiency of infection processes involved. Neck blast incidence was assessed at 12 DAI.

#### Incubation period

The incubation period is the time elapsed between spore deposition and symptom appearance [8]. Necks were observed once a day and the number of necks presenting symptoms was recorded. Neck blast symptoms started to appear at 5 DAI. Assessment was performed until 12 DAI, and incubation period was operationally defined as the time when 50% of lesions had appeared (IP50). The incubation period was estimated by linear interpolation between consecutive assessments.

#### Lesion length

Lesion length (mm) on necks was measured with a ruler at 12 DAI.

#### Sporulation intensity

Infected necks were cut from the panicle at 12 DAI, placed inside a Petri plate on double layered moist filter papers (Whatman no. 1) in order to provide a moist environment to the lesions, and incubated in dark at 25°C. After 24 hr, necks were deposited into vials containing 1 ml of 0.025% Tween20 solution. The vials were then shaken with a Vortex shaker (model: VM-2000, Digisystem Laboratory Instruments Inc., Taiwan) at full speed for 20 s to liberate the conidia. The number of spores was counted using a haemocytometer under a dissecting microscope (×40 magnification). The mean number of spores over two counts was computed for each neck. A few samples of spore suspension were stored at 4°C and counted the following day.

### Experimental Design and Data Analyses

Three independent experiments were performed to measure monocycle parameters on leaves, and three independent experiments were performed to measure monocycle parameters on necks. In all experiments, 24 isolates were used, i.e., 12 isolates originating from leaves, and 12 isolates originating from necks. In the measurements made on leaf segments, the experimental unit consisted of the pool of 4 leaf segments in a plate inoculated by a given isolate. A similar experimental design was applied for measurements made on necks, for which the experimental unit consisted of the pool of 4 plant necks inoculated by a given isolate. All experiments had a randomized complete block design with four replications. In each block, the 24 experimental units (i.e., 24 Petri plates with 4 leaf segments, and 24 pots with 4 inoculated necks for inoculations made on leaves and necks, respectively) corresponded to the 24 isolates tested, and were positioned randomly after inoculation.

Mixed model analyses of variance were conducted to assess the effect of origin of isolates on the monocyclic parameters individually on leaves and necks. Analyses were conducted using the MIXED procedure of the statistical software SAS (Cary, North Carolina, USA), where isolates were nested within origin (neck or leaf), and where isolates, experiments, and replications were considered random factors, and the only fixed factor was the origin of isolate.

The effect of isolate origin on all components of aggressiveness on leaves and necks was first analyzed by a multiple analysis of variance (MANOVA) to test the effect of isolate origin on all eight variables measured to characterize aggressiveness of the isolates on leaves and necks. A stepwise discriminant analysis was then conducted to identify the components of aggressiveness contributing most to the differences between the two groups of isolates (originating from leaves: LO-isolates or necks: NO-isolates). In both analyses, the means per isolate over the three experiments of the 24 isolates were used. Both analyses were performed using SYSTAT 13.

The relationships between the monocyclic parameters measured on leaves and necks were assessed by examining the correlation coefficients for all pairs of variables, using the means per isolate over the three experiments of the 24 isolates. Association between the different variables was further explored using a principal component analysis.

## Results

### Effect of Isolate Origin on Aggressiveness Components on Leaves

**Figure 1:**
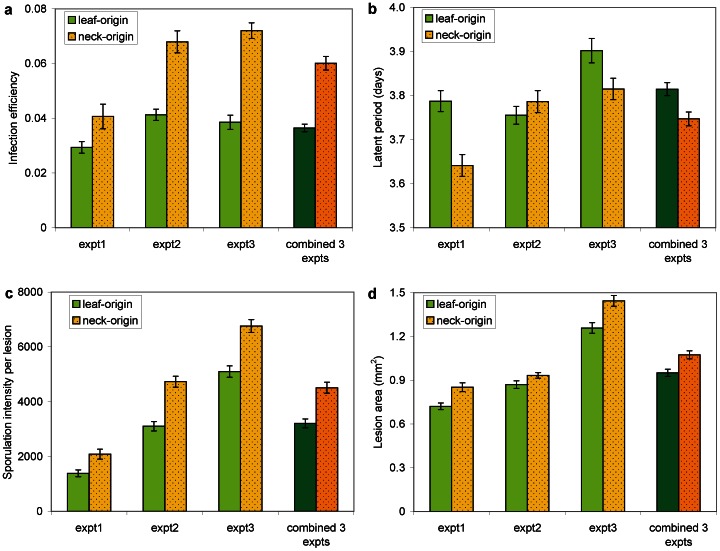
Aggressiveness components on rice leaves of *Magnaporthe oryzae* isolates originating from rice leaves and necks. In each experiment, each bar represents the mean over 48 measurements made: 12 isolates and 4 replications per isolate. The error bars represent the standard error of the means. In each graph, the three first pairs of bars represent results obtained in each of three independent experiments, and the last pair of bars represents the results obtained from the three combined experiments.

The different components of aggressiveness measured were in general larger for NO-isolates than for LO-isolates (Fig. 1). Infection efficiency ranged from 0.03 to 0.07, and was about 30% and 50% higher in NO- than LO-isolates for the first and two last experiments, respectively (Fig. 1a). LP50 ranged from 3.65 to 3.90 days. LP50 was larger for LO- than NO-isolates in the first and third experiments, indicating that LO-isolates were less aggressive on leaves than NO-isolates. LP50 for LO-isolates was however lower than for NO-isolates in the second experiment (0.03 day), but when the three experiments were combined, the mean LP50 value for LO-isolates was numerically larger than for NO-isolates, with a difference of 0.06 day (Fig. 1b). Sporulation intensity per lesion was lowest in experiment 1 and highest in experiment 3 (Fig. 1c), and ranged between 1,400 and 6,800 spores per lesion. In all experiments, sporulation intensity was larger for NO- than for LO-isolates. As for sporulation intensity, lesion area was lowest in experiment 1 and largest in experiment 3 (Fig. 1d), and was always larger for NO-isolates than for LO-isolates. Lesion area ranged between 0.72 and 1.44 mm^2^. The mixed model analyses of variance indicated that the effect of organ origin was significant (*P*<0.05) for all components of aggressiveness except LP50 (Table 1).

**Table 1:** Results from the mixed model analyses of variance of the effect of isolate origin (leaf or neck) on the components of aggressiveness on leaves and necks in rice blast.

Component of aggressiveness	Aggressiveness on leaves		Aggressiveness on necks	
	F	Pr>F	F	Pr>F
Infection efficiency/disease incidence^1^	6.37	0.032	3.67	0.068
Latent period/incubation period^2^	1.65	0.32	1.52	0.23
Sporulation intensity	12.45	0.015	2.44	0.20
Lesion area/lesion length^3^	9.38	0.043	521.44	<0.0001

1 infection efficiency on leaves, disease incidence on necks.

2 latent period on leaves, incubation period on necks.

3 lesion area on leaves, lesion length on necks.

### Effect of Isolate Origin on Aggressiveness Components on Necks

**Figure 2:**
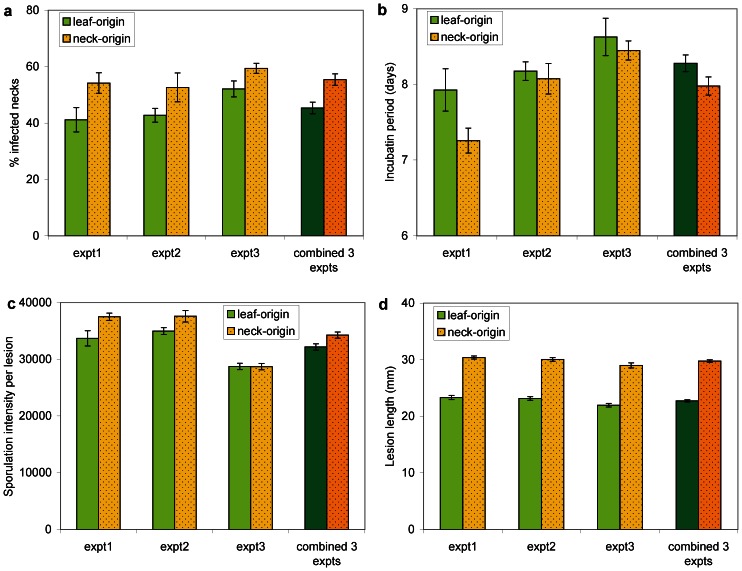
Aggressiveness components on rice necks of *Magnaporthe oryzae* isolates originating from rice leaves and necks. In each experiment, each bar represents the mean over 48 measurements made: 12 isolates and 4 replications per isolate. The error bars represent the standard error of the means. In each graph, the three first pairs of bars represent results obtained in each of three independent experiments, and the last pair of bars represents the results obtained from the three combined experiments.

Similarly to results obtained on leaves, the different components of aggressiveness on necks were larger for NO-isolates than for LO-isolates (Fig. 2). The percentage of infected necks ranged from 41% to 59% and difference between LO- and NO-isolates ranged between 7% and 13% among the three experiments (Fig. 2a). The incubation period ranged between 7.2 and 8.6 days, and the difference between isolate origins was larger in experiment 1 than in the other two (Fig. 2b). Sporulation intensity ranged between 28,700 and 37,600 spores per lesion. Difference between LO- and NO-isolates was observed in experiments 1 and 2, but not in experiment 3 (Fig. 2c). Lesion length ranged between 22 and 30 mm, and a difference of 6–7 mm between LO- and NO-isolates was observed in all three experiments (Fig. 2d). The results obtained from the mixed model ANOVAs indicated that the effect of isolate origin was significant (*P*<0.0001) only for lesion length (Table 1). The other components of aggressiveness were associated with large standard errors of the mean, precluding the detection of the effect of isolate origin.

### Effect of Isolate Origin on Aggressiveness on Leaves and Necks

The MANOVA of the effect of isolate origin on the components of aggressiveness on leaves and necks yielded a Wilks' Lambda value of 0.019 with 8 and 15 degrees of freedom, and corresponded to an F-ratio of 97.5, associated with a significant probability (*P*<0.001).

The stepwise discriminant analysis retained five variables to discriminate the origin of isolates: infection efficiency, lesion area, and sporulation intensity on leaves; and incubation period, and lesion length on necks. These five variables allowed classifying correctly all isolates according to their origin.

### Relationships between Aggressiveness Components on Leaves and Necks

The monocyclic parameters that were most correlated were infection efficiency, sporulation intensity, and lesion area on leaves (*r* >0.8, *P*<0.0001; Table 2). These parameters were positively correlated with lesion length and sporulation intensity on necks (*r* >0.56, *P*<0.001; Table 2). The latent period on leaves was negatively correlated (*P*<0.05) with the three other parameters measured on leaves, as well as with lesion length on necks. The incidence on necks, lesion length and sporulation intensity on necks were positively correlated (*P*<0.05), and the incubation period on necks was negatively correlated with the other parameters measured on necks, as well as with infection efficiency on leaves (*P*<0.05).

**Table 2:** Pearson correlation coefficients between monocycle variables measured.

Monocycle variable^1^	IE	LP50	LSA	SIL	INC	IP50	LLN	SIN
**IE**	1	−0.58**	0.81***	0.93***	0.27	−0.43*	0.56**	0.63**
**LP50**		1	−0.45*	−0.56**	−0.33	0.21	−0.47*	−0.25
**LSA**			1	0.93***	0.05	−0.22	0.63**	0.66**
**SIL**				1	0.23	−0.34	0.69**	0.71**
**INC**					1	−0.64**	0.44*	0.49*
**IP50**						1	−0.45*	−0.57**
**LLN**							1	0.47*
**SIN**								1

1 IE: infection efficiency on leaves; LP50: latent period on leaves; LSA: lesion area on leaves; SIL: sporulation intensity per lesion on leaves; INC: neck blast incidence; IP50: incubation period on necks; LLN: lesion length on necks; SIN: sporulation intensity per lesion on necks.

* : *P*<0.05;

** : *P*<0.01;

*** : *P*<0.0001.

**Figure 3:**
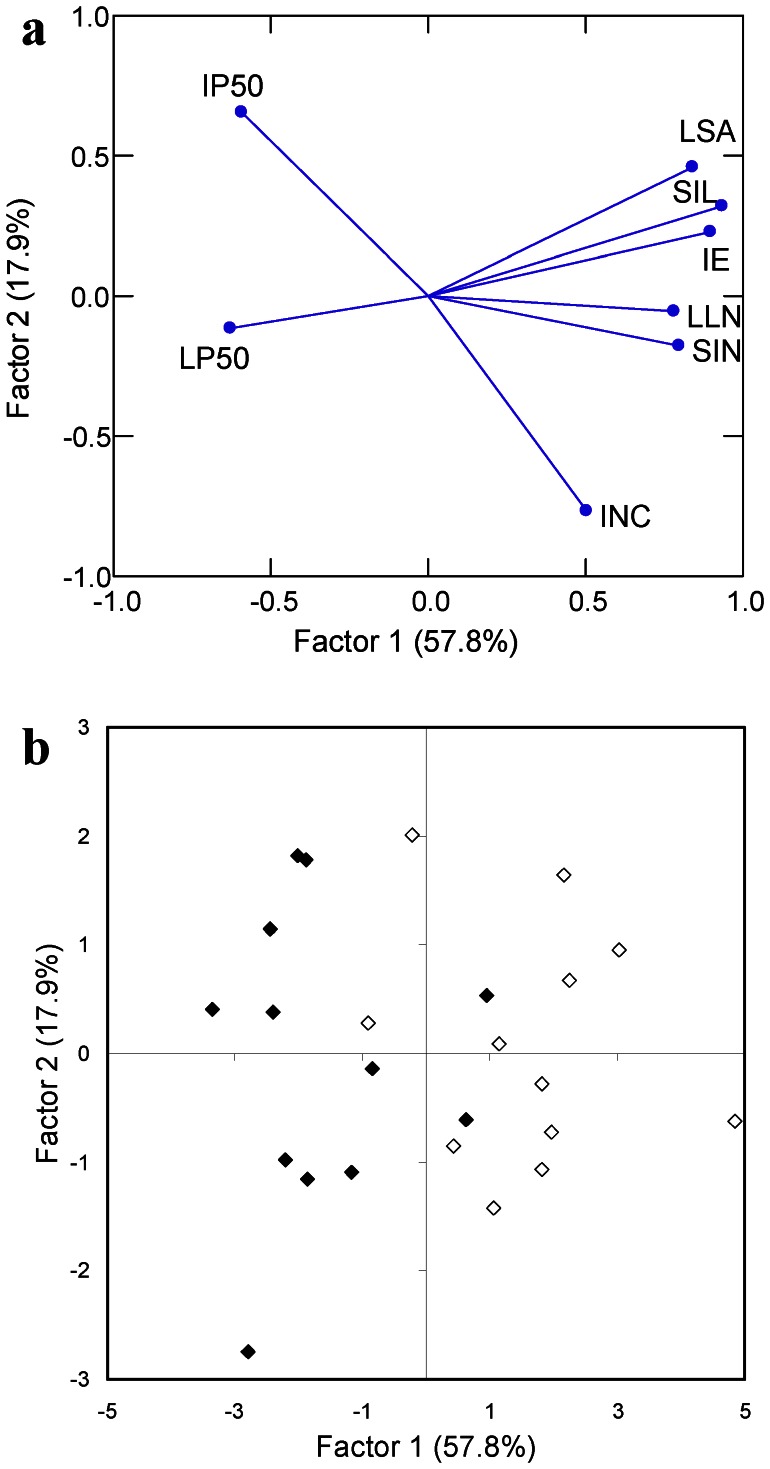
Principal component analysis from components of aggressiveness of *Magnaporthe oryzae* isolates on leaves and necks. a: component loadings of the component of aggressiveness. IE: infection efficiency on leaves; LP50: latent period on leaves; LSA: lesion area on leaves; SIL: sporulation intensity per lesion on leaves; INC: neck blast incidence; IP50: incubation period on necks; LLN: lesion length on necks; SIN: sporulation intensity per lesion on necks. b: factor scores of the 24 *Magnaporthe oryzae* isolates displayed on the two first axes. Black diamonds: isolates originating from leaves; white diamonds: isolates originating from necks.

The two first axes generated by the principal component analysis captured the majority of the variance, accounting for 57.8 and 17.9% of the variance, respectively (Fig. 3a). The first axis was characterized by two groups of variables, one including variables with positive correlation (infection efficiency, sporulation intensity, and lesion area on leaves; disease incidence, lesion length and sporulation intensity on necks), and the other one with negative correlation (latent period on leaves and incubation period on necks). On the second axis, incidence and incubation period on necks were the two most separated variables. The principal component analysis allowed positioning the 24 isolates according to the two first components (Fig. 3b). The isolates originating from necks and leaves were mainly differentiated according to the first axis. Isolates originating from the necks were positioned in majority on the right part of the graph, corresponding to larger values of infection efficiency, sporulation intensity, and lesion area on leaves, and lesion length and sporulation intensity on necks, and low values of latency period on leaves, whereas the isolates originating from leaves were mainly localised on the left part of the graph.

## Discussion

The different analyses conducted (analyses of variance, MANOVA, principal component analysis, and discriminant analysis) indicate that isolates originating from necks were more aggressive than isolates originating from leaves, when aggressiveness components were measured both on leaves and necks. This result can be explained by the hypothesis that amongst the population of *M. oryzae* isolates infecting leaves, the more aggressive ones have a higher probability to infect necks. This result also suggests that there is no specialization within the pathogen population with respect to the type of organ infected: if such were the case, isolates originating from a given organ would have been more aggressive on the organ of origin than on the other. Difference in life-cycle traits between isolates originating from leaves and necks further indicate that changes in population take place over the course of blast dual epidemics developing within a cropping season. Disease transmission from leaves to necks may represent a population bottleneck, whereby the more aggressive isolates are associated with a higher probability to infect necks, leading to a shift in pathogen population. Whether this hypothesis generated from phenotypic characteristics corresponds to genetic shifts within the pathogen population remains to be tested. Population bottlenecks and their implication on population evolution have been documented for plant viruses across different stages of their life cycle. For example, population bottlenecks occur during systemic movement within the host as shown in the case of wheat streak mosaic virus [12], and during disease transmission between hosts as in the case of tobacco mosaic virus [13].

The ranges in the aggressiveness components obtained in this work corresponded to ranges obtained in previous studies conducted under similar conditions. Latent period on leaves between 3.7 and 3.9 days was found, which is close to reported values (4 days) [14]. Sporulation intensity per lesion on leaves ranged between 2,000 and 6,000, which is a range similar to ranges reported [15]. Lesion size at 5 DAI in our study ranged between 0.7 and 1.4 mm^2^. This range was lower than values found earlier [10], but which corresponded to lesion size at 9 DAI. Incubation periods for neck blast were estimated to 9–10 days [16], and 7–9 days [17], which are close to the results obtained in this study. Lesion lengths on necks ranged between 20 and 30 mm, which are close to the values obtained earlier [17]. Sporulation intensity on necks ranged between 25,000 and 40,000 spores per lesion in our study, which corresponds to ranges found at 15 DAI [18] (20,000–80,000 spores per lesion).

Correlations between aggressiveness components on leaves (infection efficiency, latent period, sporulation intensity and lesion area) were significant (*P*<0.05) in all cases, with positive correlations between all variables except latent period, which was negatively correlated with all other variables. A short latent period corresponds to high aggressiveness, which in turn corresponds to large values of the other components of aggressiveness. This finding therefore indicates that the different stages of the life cycle, from infection to colonization and production of propagules, are correlated in terms of process efficiency. The same conclusion can also be drawn in the case of the components of aggressiveness on necks, which were all significantly (*P*<0.05) correlated. Correlation between components of aggressiveness has been observed on other pathosystems such as potato late blight [19] and wheat powdery mildew [20].

Correlation between components of aggressiveness on leaves and necks were also detected: lesion length and sporulation intensity on necks were significantly (*P*<0.05) correlated with all components of aggressiveness on leaves except in one case (latent period on leaves and sporulation intensity on necks). This result indicates that processes involved in the non-dispersive phase of the disease cycle on leaves and necks are governed by similar mechanisms, at least in susceptible genotypes. However, when resistance is expressed, despite the positive correlation between the level of resistance on leaves and the level of resistance on necks, several rice genotypes showed a differential expression depending on the organ [5], [7], thus indicating a differentiation in the mechanisms involved in the resistance in both types of organs.

Our results indicate that blast isolates originating from leaves can infect necks. Management of the disease in order to reduce disease intensity on necks (which causes high blast losses in tropical Asia as opposed to leaf blast; [6]) may therefore include tools limiting epidemics on leaves, at the field or at the landscape scales. This implies management strategies of a whole pathosystem, including a range of variable and genetically flexible isolates, resistances of different kinds [21], and different types of injuries.
